# Comparative Effectiveness of Hepatic Artery Based Therapies for Unresectable Colorectal Liver Metastases: A Meta-Analysis

**DOI:** 10.1371/journal.pone.0139940

**Published:** 2015-10-08

**Authors:** Anthony J. Zacharias, Thejus T. Jayakrishnan, Rahul Rajeev, William S. Rilling, James P. Thomas, Ben George, Fabian M. Johnston, T. Clark Gamblin, Kiran K. Turaga

**Affiliations:** 1 Division of Surgical Oncology, Department of Surgery, Medical College of Wisconsin, Milwaukee, Wisconsin, United States of America; 2 Division of Interventional Radiology, Department of Radiology, Medical College of Wisconsin, Milwaukee, Wisconsin, United States of America; 3 Division of Hematology and Oncology, Department of Medicine, Medical College of Wisconsin, Milwaukee, Wisconsin, United States of America; ISMETT-UPMC Italy/ University of Catania, ITALY

## Abstract

**Background:**

Patients with unresectable Colorectal Liver Metastases (CRLM) are increasingly being managed using Hepatic Artery Based Therapies (HAT), including Hepatic Arterial Infusion (HAI), Radioembolization (RE), and Transcatheter Arterial Chemoembolization (TACE). Limited data is available on the comparative effectiveness of these options. We hypothesized that outcomes in terms of survival and toxicity were equivalent across the three strategies.

**Methods:**

A meta-analysis was performed using a prospectively registered search strategy at PROSPERO (CRD42013003861) that utilized studies from PubMed (2003–2013). Primary outcome was median overall survival (OS). Secondary outcomes were treatment toxicity, tumor response, and conversion of the tumor to resectable. Additional covariates included prior or concurrent systemic therapy.

**Results:**

Of 491 studies screened, 90 were selected for analyses—52 (n = 3,000 patients) HAI, 24 (n = 1,268) RE, 14 (n = 1,038) TACE. The median OS (95% CI) for patients receiving HAT in the first-line were RE 29.4 vs. HAI 21.4 vs. TACE 15.2 months (p = 0.97, 0.69 respectively). For patients failing at least one line of prior systemic therapy, the survival outcomes were TACE 21.3 (20.6–22.4) months vs. HAI 13.2 (12.2–14.2) months vs. RE 10.7 (9.5–12.0). Grade 3–4 toxicity for HAT alone was 40% in the HAI group, 19% in the RE group, and 18% in the TACE groups, which was increased with the addition of systemic chemotherapy. Level 1 evidence was available in 5 studies for HAI, 2 studies for RE and 1 for TACE.

**Conclusion:**

HAI, RE, and TACE are equally effective in patients with unresectable CRLM with marginal differences in survival.

## Introduction

Colorectal cancer is the second leading cause of cancer-related deaths in the United States [1]. In 2009, 136,717 people were diagnosed with colorectal cancer in the US with 51,848 deaths resulting from the disease.[1,2] Liver is the most common site of colorectal metastases, with approximately 50% of those diagnosed developing metastases, either synchronous or metachronous within 2 years after primary diagnoses.[3] In the treatment of colorectal liver metastases (CRLM), less than 25% of patients become eligible for surgical resection which is the only potentially curative option.[4] [5]

For unresectable CRLM, systemic therapy has been shown to achieve an overall survival of 15–20 months and deaths are mostly attributed to hepatic progression.[6–8] In an effort to improve survival, hepatic arterial therapies (HAT) were developed with the understanding that hepatic tumors primarily utilize the hepatic artery for blood supply, as opposed to the native liver, that relies heavily on portal circulation.[2,9–11] In comparison to intravenous chemotherapy and external beam radiation therapy, the direct approach also allows for higher tolerable dose to the target disease.

The three commonly used local-regional strategies are hepatic arterial infusion (HAI), radioembolization (RE), and transcatheter chemoembolization (TACE). HAI delivers antimetabolites to the liver via an intra-arterially placed catheter connected to a subcutaneous port/pump. Catheter placement is typically done during surgical resection of the primary tumor, or percutaneously via the femoral artery.[12] Regimens for chemotherapy vary from weekly infusions over the course of a few hours to 14 day continuous infusion every four weeks until disease progression, or toxicity/complications from the pump/catheter forcing treatment termination.[13,14] TACE combines various high dose chemotherapy agents in an emulsion with lipiodol with subsequent embolization, or the use of drug eluting beads loaded with Irinotecan (DEBIRI). Radioembolization (RE) uses Yttrium-90 beta emitting microspheres percutaneously introduced into the hepatic artery. Two types of microspheres are available: resin (SIR-Spheres, Sirtex Medical, Sydney, Australia) and glass (TheraSphere, BTG Farnham, UK).

We hypothesized that outcomes in terms of survival and toxicity were equivalent across the three strategies of HAI, RE and TACE.

## Methods

### Search strategy

The study protocol was prospectively registered with the PROSPERO International prospective register of systematic review**s** (Study ID: CRD42013003861). A systematic search was conducted in PubMed database to identify studies (May 2003—June 2013) reporting the outcomes of HAI, TACE, or RE in the treatment of unresectable CRLM (S1 Text).

### Outcomes

#### Primary outcome

Median overall survival (OS)

#### Secondary outcomes

1. Treatment related toxicity–National Cancer Institute (NCI) Common Toxicity Criteria for Adverse Events or World Health Organization (WHO) criteria
2. Tumor response–Response Evaluation Criteria in Solid Tumors (RECIST), modified RECIST (mRECIST), WHO criteria
3. Conversion to surgically resectable

### Eligibility Criteria

#### Inclusion criteria

1. Randomized control trials or observational studies
2. Human studies
3. Published in PubMed in English language
4. Published between May 2003—June 2013
5. Patients with synchronous or metachronous CRLM treated with HAT including: HAI, TACE, or RE
6. Reporting primary outcome of interest

#### Exclusion Criteria

1. Case series (<10 patients) or case reports
2. Studies on primary liver neoplasm
3. Metastases from anal primary/primary other than colorectal
4. Duplicate studies unless if they presented unique information (Example—analysis in pretreated patients)
5. Patients with resectable metastases
6. Concurrent treatments besides systemic chemotherapy

Mixed cohort studies were included if separate analysis was reported for patients with unresectable CRLM. Uncertainties were resolved by consensus amongst researchers (AZ, TJ and KT).

### Response and Complications Assessment

Overall response rates (complete and partial) were abstracted and graded by WHO, RECIST, or mRECIST guidelines.[15–17] Toxicity rates were calculated as the total grade 3–4 events out of the total number of patients from the studies reporting a total number of complications using WHO or NCI Common Toxicity Criteria for Adverse Events. Organ specific complication rates were calculated only from articles that described them specifically. Missing or inadequate data were evaluated by imputation, and excluded for the sub-analysis if no dramatic differences were noted in effect size.

### Statistical Analysis

Statistical calculations were performed with STATA software Version 12.1 (StataCorp, Texas, USA). Meta-analysis were performed using the random effects modeling techniques using STATA subroutines *met*an and meta-regression using the *meta-reg* subroutine. Forest plots were created for visual depiction of data. Publication bias was explored using funnel plots, and inverse variance weighting was utilized for performing meta-analyses.[18] Standard error (SE) was calculated from 95% confidence interval/range as available from the studies.[19] Missing standard errors were imputed using the average of the SE of reported studies. Missing covariates such as number of lesions, line of chemotherapy and demographics Mean age was converted to a median, if median age was not reported, using equations based on the study sample size.[19] Additional covariates were use of prior/ concurrent systemic chemotherapy. Patients who received HAT as first line therapy were classified as chemo naïve and those that received prior systemic treatment were classified as pretreated. We estimated 95% confidence interval where they were not available.[20]

## Results

**Fig 1:**
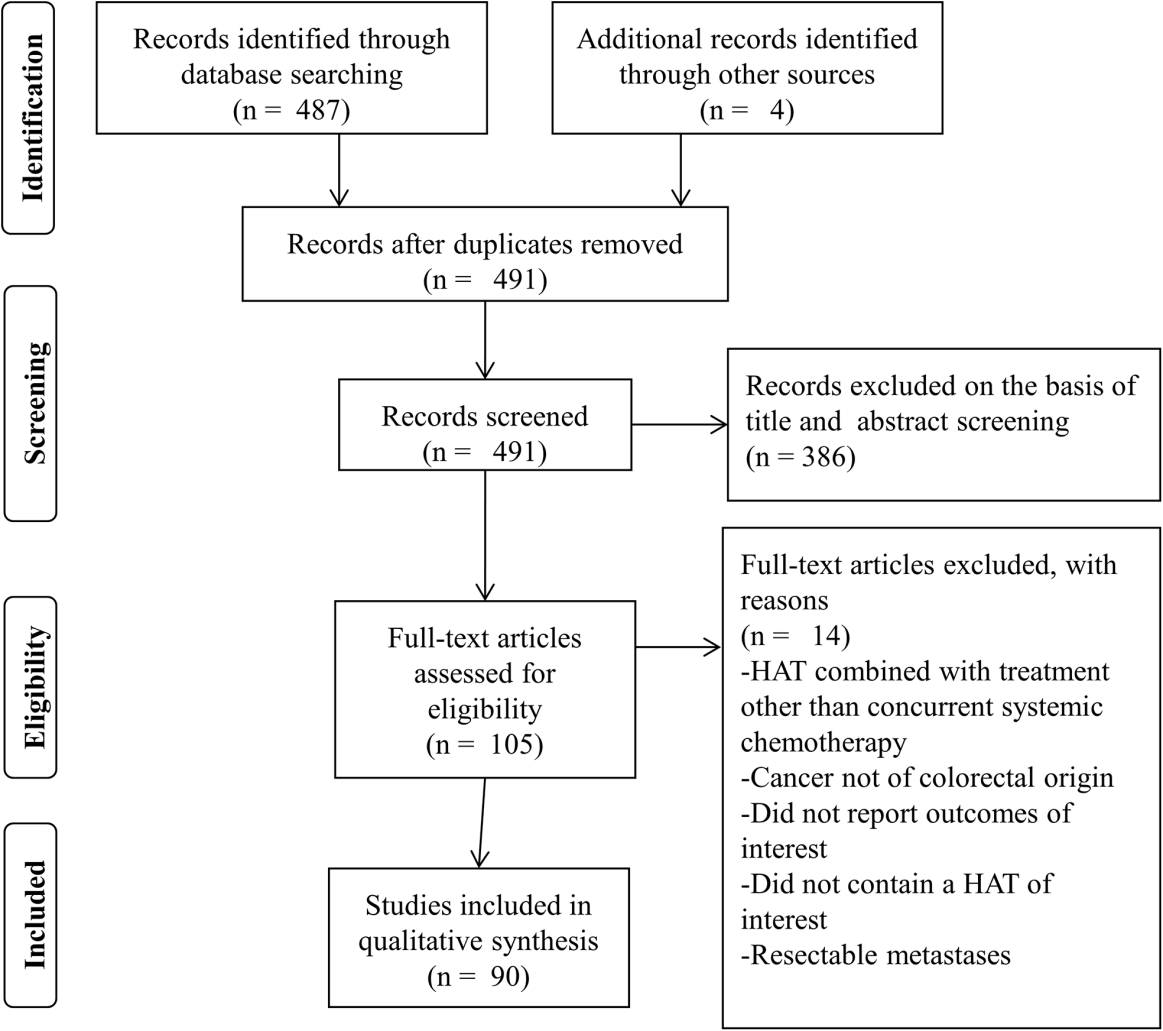
PRISMA Flow Chart Depicting Stages of Article Selection [21]. HAT hepatic artery based therapy.

The search strategy resulted in 487 articles of which 90 were eligible for analysis (Fig 1). The individual studies on HAI, RE, or TACE used for data extraction are described in S1, S2 and S3 Tables (online only), respectively.

### Quality Appraisal

Of the studies included in the analysis, the majority were prospective registry studies -HAI 52%, RE 42%, and TACE 50%. There were a few randomized controlled trials (RCT) -5 (9%) for HAI, 2 (8%) for RE, and 1 (7%) for TACE. Quality appraisal of the eligible studies is represented in S1 Fig (online only).

### Patient Demographics

Demographic summary of patients included in the study (90 studies, n = 5306) is described in Table 1. Of these, 52 studies (n = 3000 patients) examined HAI, 24 (n = 1268) RE, and 14 (n = 1038) TACE. The median ages and gender distribution were comparable. Of those receiving RE and TACE, 94% and 91% of the subjects respectively, had received at least one prior line of chemotherapy for metastatic disease vs. 42% in HAI. Extra-hepatic disease (EHD) prior to treatment was present in 36% of those receiving RE vs. 18% with HAI vs. 13% with TACE. The most common site of primary was the colon in 71% HAI, 85% RE, and 68% TACE patients.

**Table 1:** Demographic Summary of the Studies Included for Meta-analysis.

Category	HAI	RE	TACE
Articles	52	24	14
Patients	3000	1268	1038
Average median age of patients	61±4 (2551)	62±3 (1222)	62 ±4 (990)
Male (%)	66% (2197)	65% (1226)	63% (1018)
Pretreated	42% (2089)	94% (1133)	91% (893)
Extra-hepatic disease	18% (2002)	36% (1018)	13% (1084)
Colon origin (%)	71% (1445)	85% (368)	68% (541)

HAI hepatic arterial infusion; RE radioembolization; TACE transcatheter chemoembolization

### Survival Outcomes

Survival outcomes are described in Table 2. When stratified by prior treatment, median OS of those receiving HAT as a first line option vs. those failing at least one prior line of chemotherapy was greater in the HAI and the RE groups, although a similar effect was not seen with TACE. Median OS of HAI in the first line setting was 18.8 (13.8–23.9) months without concurrent systemic therapy vs. 21.0 (18.8–23.2) months with its addition. No studies were available analyzing RE or TACE alone in the first line setting. In pretreated patients treated with HAT alone, median OS were TACE 21.3 (20.4–22.2) months vs. HAI 12.6 (11.5–13.7) months vs. RE 10.9 (9.3–12.5) months (df = 2, p-value < 0.001). When used in conjunction with concurrent systemic chemotherapy, median OS in pretreated patients were HAI 13.9 (4.4–23.3) and 13.0 (8.0–18.0) months in RE (df = 1, p-value = 0.874). No survival data was available for pretreated patients receiving TACE with concurrent systemic chemotherapy.

**Table 2:** Survival Outcomes (median survival in months) of HAI, RE and TACE Stratified into Appropriate Subgroups for Comparison.

	Median Survival (months)		
	HAI	RE	TACE
Overall (95% CI)	16.0 (14.7–16.4)	11.4 (10.2–12.6)	21.0 (20.6–22.4)
Overall—Chemo naïve (95% CI)	21.4 (19.4–23.4)	29.4 (23.4–35.4)	15.2^a^
Overall–Pretreated (95% CI)	13.2 (12.2–14.2)	10.7 (9.5–12.0)	21.3 (20.6–22.4)
HAT Alone (95% CI)	13.3 (12.3–14.3)	10.5 (9.2–11.8)	21.1 (20.2–22.0)
HAT + Sys (95% CI)	22.5 (20.4–24.5)	19.7 (15.9–23.6)	15.9 (14.8–17.2)
HAT Alone in Chemo naïve (95% CI)	18.8 (13.8–23.9)	-	-
HAT + Sys in Chemo naïve (95% CI)	21.0 (18.8–23.2)	29.4 (23.4–35.4)	15.2^a^
HAT alone in pretreated (95% CI)	12.6 (11.5–13.7)	10.9 (9.3–12.5)	21.3 (20.4–22.2)
HAT +Sys in Pretreated (95% CI)	13.9 (4.4–23.3)	13.0 (8.0–18.0)	-

^a^ 1 study available for survival data—no data available

HAT—hepatic artery based therapy; HAI–Hepatic artery infusion. RE- Radioembolization TACE—Transcatheter Arterial Chemoembolization. Sys—systemic chemotherapy

### Response Outcomes

Response rates (partial and complete) for the three treatment strategies are represented in Table 3. When including all patients receiving HAT regardless of concurrent systemic chemotherapy, response rates were HAI 48 (95% CI 42–55)% vs. RE 36 (25–47)% and TACE 29 (14–43)%.

**Table 3:** Summary of Response Rates (Complete and Partial) of HAI, RE and TACE Stratified into Appropriate Subgroups for Comparison.

	Response Rates		
	HAI	RE	TACE
Overall (95% CI)	48 (42–54)	36 (25–47)	29 (14–43)
Overall—Chemo naïve (95% CI)	52 (39–65)	90 (88–92)	90^a^
Overall–Pretreated (95% CI)	35 (24–45)	32 (24–39)	28 (7–48)
HAT Alone (95% CI)	47 (38–55)	35 (24–45)	22 (4–41)
HAT + Sys (95% CI)	52 (41–64)	61 (16–100)	46 (22–70)
HAT Alone in Chemo naïve (95% CI)	53 (20–90)	-	-
HAT + Sys in Chemo naïve (95% CI)	51 (38–63)	90 (88–92)	90^a^
HAT alone in pretreated (95% CI)	37 (20–55)	34.5 (24–45)	24 (0–49)
HAT +Sys in Pretreated (95% CI)	36 (14–58)	30 (10–48)	-

- no data available.

^a^ 1 study available for response data

HAT—hepatic artery based therapy; HAI–Hepatic artery infusion. RE- Radioembolization TACE—Transcatheter Arterial Chemoembolization. Sys—systemic chemotherapy

### Conversion to Resectable

When analyzing conversion to resectable in all studies, regardless of line of therapy and extrahepatic disease, HAI had the highest conversion rate 15 (95% CI 13–17)% followed by TACE 4 (2–7)% and RE 2 (1–4)%. Analysis done by prior treatment revealed higher conversion rates in the first-line setting vs. pretreatment for metastatic disease–HAI 13 (9–18%) vs. 8 (5–13)%, RE 10 (2–29)% vs. 1 (0–3)%), and TACE 40 (12–74)% vs. 4 (2–9)%). In the first line setting, HAI with concurrent systemic chemotherapy was superior to HAI alone 24 (17–31%) vs. 7 (3.3–13)%, respectively).

### Treatment Toxicity

**Fig 2:**
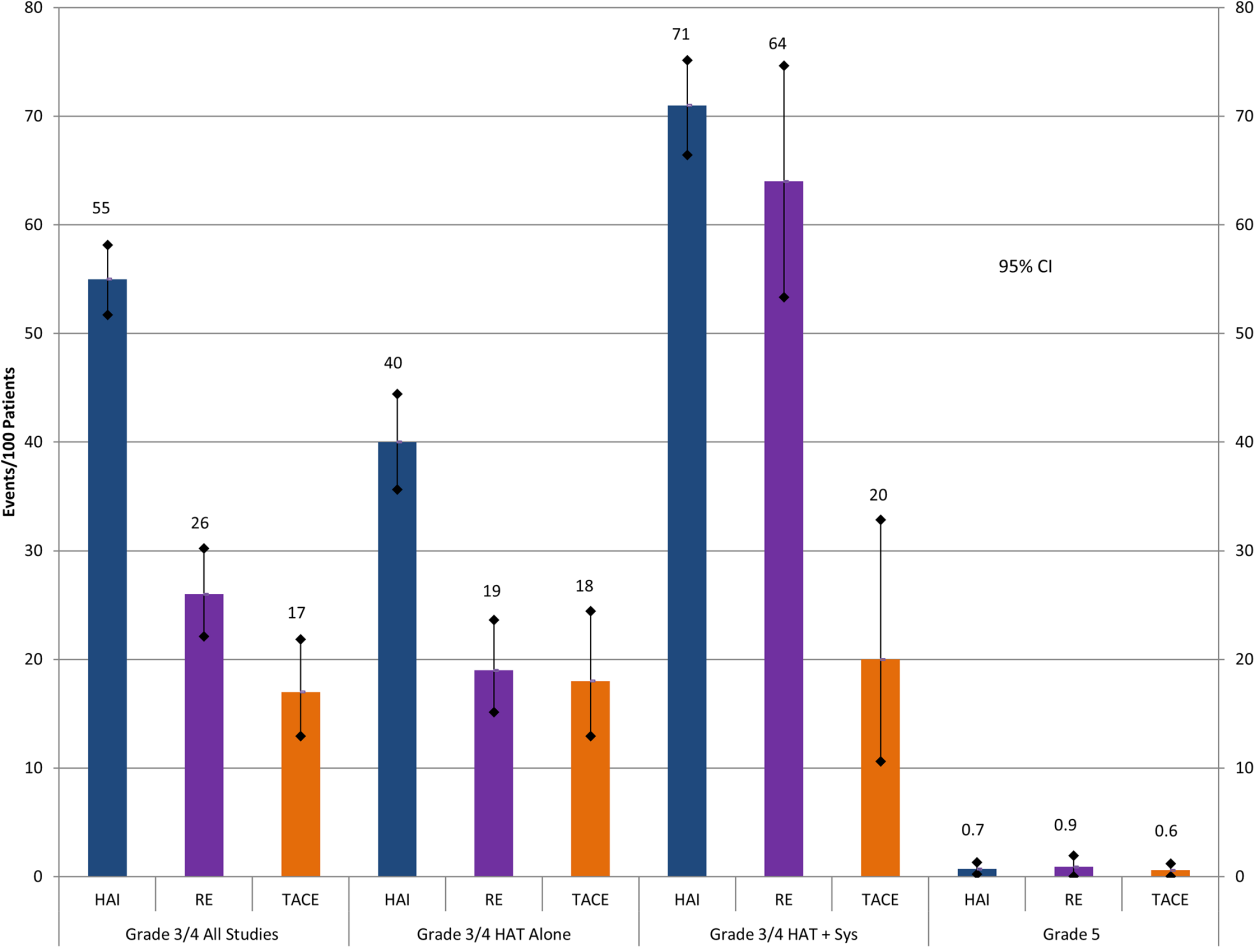
Distribution of Toxicity (Events per 100 Patients) Reported Across HAI, RE and TACE. HAI hepatic arterial infusion; RE radioembolization; TACE transcatheter chemoembolization; HAT hepatic artery based therapy; Sys concurrent systemic chemotherapy.

Combined grade 3–4 toxicities for each treatment are graphically represented in Fig 2. HAI was associated with the highest incidence of grade 3–4 toxicity per patient at 55 (52–58)%, followed by RE 26 (22–30)% and TACE 17 (13–22)%. Toxicity rates increased when HAT was combined with systemic therapy compared to use without concurrent systemic therapy. Common organ specific complications are highlighted in S4 Table (online only). In HAI group, treatment was terminated due to technical complications with the pump/catheter in 11% of patients. These included catheter displacement, catheter thrombosis/occlusion, and hepatic artery thrombosis/occlusion. Mortality resulting from treatment was less than 2% in all strategies.

### Multivariate analysis

The difference between mortality rates between HAI and TACE was (-0.006 (-0.34 to 0.33), p = 0.97) and HAI and RE was (0.045 (-0.17 to 0.26), p = 0.69). The inferences were unchanged after adjusting for median age, and previous number of chemotherapy lines.

## Discussion

In a comprehensive analysis of the available literature on the role of HAT in the management of patients with unresectable CRLM, this study reports the effectiveness of HAI, TACE and RE with marginal differences. In the first line setting, RE appears to have marginal advantages over other modalities, while TACE appeared to be more effective in second line settings. HAI infusion therapy has the largest body of quality scientific evidence and demonstrates consistent benefit in survival, and a slightly higher conversion to resectability in the pretreated setting. These therapies have become valuable adjuncts in the management of patients and in conjunction with systemic chemotherapy can significantly prolong the survival. Making the appropriate choice of treatment based on patient specific factors is difficult and in the era of shared decision making, the ability to clearly present the data may facilitate such discussions.

The evidence for HAI is the strongest with 5 large randomized trials demonstrating its efficacy. Our study clearly shows an improvement in survival with addition of systemic therapy, which was seen even in pre-treated patients. The response rates and conversion to resectability for HAI were comparable to other strategies, albeit slightly higher. However, HAI had and increased cost of toxicity with a 55% grade 3–4 toxicity that patients have to endure, which could potentially lead to loss of quality of life, ability to continue with systemic therapy and economic burden. In addition to higher toxicity rates, the complications with the pump/catheter resulted in treatment termination in 11% of the HAI group. The termination rate increased from 6.5–8% with HAI alone to 20% in HAI combined with systemic chemotherapy.[22–24] Common causes for termination were catheter thrombosis/occlusion, displacement of catheter, and hepatic arterial occlusion/thrombosis. After termination of HAI, patients typically received systemic chemotherapy or palliative care. This highlights the importance of prophylactic treatment and continuous maintenance of the infusion system due to the potential negative impact of early treatment termination on the treatment outcome. In contrast, technical complications with RE and TACE are rare.

Radioembolization has been studied most in the pretreated population and continues to show a survival benefit in this setting. While the response rates can be dramatic in small studies, conversion to resectability was also respectable in the pooled analysis. This is despite higher rates of pretreatment extra-hepatic metastases observed in this patient cohort. Notably, the Grade 3–4 toxicities were also low. Long term radiation induced effects of radioembolization could not be assessed due to the relative novelty of the treatment.

Chemoembolization showed a similar response rate in small studies, but a less modest survival benefit. A rather paradoxical effect was seen with the addition of systemic chemotherapy in patients undergoing chemoembolization, with no additional survival benefit. The toxicity profile is less favorable than RE given the embolization that occurs with this technique, yet is fairly comparable. Single studies did have a significant weight on the pooled analysis and exclusion of a study led to dramatically different inferences, making this a less reliable inference.

When stratified by timing of treatment, RE provided a higher survival benefit than HAI and TACE as a first line treatment. However, only 3 studies reported on the use of RE and TACE as a first line option, with all the patients being treated with concurrent systemic chemotherapy. The survival outcome with HAI improved when combined with concurrent systemic chemotherapy. This was also reflected in a multi-center RCT that found a significantly improved median OS when HAI was combined with systemic chemotherapy (20 vs. 14 months; p = 0.003) as a first-line treatment.[25] Due to paucity of data, we were unable to determine the relationship of concurrent systemic chemotherapy with RE or TACE in the first line setting. In the pretreated setting, survival outcomes were most favorable with TACE, compared to other HAT strategies or systemic therapy alone in the second-line setting (8.6–12.9 months).[26–28] It should be noted that patients analyzed in the pretreated population of this study often received multiple lines of prior treatment. The introduction of temporal bias with improving systemic chemotherapy including biologic agents such as bevacizumab and anti-EGFR therapies, might favor improved survival outcomes for more recent therapies. Conversion to resection could also have had an impact on the survival but could not be analyzed due to insufficient data.

Studies included in our study differed in the proportion of extra-hepatic disease included in the treated arms. Patients undergoing RE were more likely to have extra-hepatic disease than those undergoing HAI or TACE therapy (Table 1). Additionally response rates were measured at differing time points by differing scales including the WHO scale, the RECIST scale and the modified RECIST. This makes drawing inference about response rate difficult, and we preferred conversion to resectability as a more robust measure of response. In pre-treated patients, HAI led to conversion rates of about 8% compared to 4% TACE and 1% RE. However, as shown in Table 1, RE had a higher rate of extrahepatic disease, which may be a contraindication to hepatic resection and thus resulting in a lower conversion rate.

### Limitations

Limitations for the present study include heterogeneity of patient selection, treatment protocols, and outcomes reporting. This heterogeneity disallows further analysis, such as meta-regression by treatment and patient characteristics. Many studies did not report demographics sufficiently, further increasing the inability to make adequate comparison. These limitations are inherent to evidence synthesis from observational studies. We did explore the heterogeneity of the results through subgroup analyses as reported in the results.

## Conclusion

HAT can potentially improve the outcome for patients with unresectable CRLM in both the first line and second line settings. The outcomes were shown to improve with the use of concurrent systemic chemotherapy. To date, the use of RE and TACE has mainly been studied as a second-line option, however improved response rates and survival has been shown in the first-line setting. Toxicity varies across the different strategies and should be an important factor in the selection criteria of patients for HAT.

## Supporting Information

S1 Fig Graphical Representation of Quality of Studies Included in the Meta Analysis. (TIF)

S1 Table Summary of Hepatic Arterial Infusion Articles Included. Sys systemic chemotherapy; EHD extrahepatic disease; OS overall survival; 5-FU Fluorouracil; Ox Oxaliplatin; Cis Cisplatin; Iri Irinotecan; Mit C Mitomycin C; LV Leucovorin; Pir Pirarubicin; Epi Epirubicin; UFT Tegafur-uracil; FUDR Floxuridine; Dox Doxorubicin. ^a^ Mean age (in years) ^b^Response = Complete Response + Partial Response.
(DOCX)

S2 Table Summary of Radioembolization Articles Included. Sys systemic chemotherapy; EHD extrahepatic disease; OS overall survival. ^a^ Mean age (in years) ^b^Subsequent HAI ^c^Response = Complete Response + Partial Response.
(DOCX)

S3 Table Summary of Transcatheter Arterial Chemoembolization Articles Included. Sys systemic chemotherapy; EHD extrahepatic disease; OS overall survival; 5-FU Fluorouracil; Cis Cisplatin; Iri Irinotecan; Mit C Mitomycin C; Gem Gemcitabine; Dox Doxorubicin. ^a^ Mean age (in years) ^b^Response = Complete Response + Partial Response.
(DOCX)

S4 Table Summary of Specific Grade 3–4 Toxicity (Events per Patient) for HAI, RE and TACE Stratified into Appropriate Subgroups for Comparison. HAI hepatic arterial infusion; RE radioembolization; TACE transcatheter chemoembolization; HAT hepatic artery based therapy; Sys concurrent systemic chemotherapy; ALP Alkaline Phosphatase.
(DOCX)

S1 Text Search strategy of systematic review. (DOCX)
