# Supplementary material for: Comparative Effectiveness of Hepatic Artery Based Therapies for Unresectable Colorectal Liver Metastases: A Meta-Analysis
Source: PLoS One. 2015 Oct 8;10(10):e0139940. doi: 10.1371/journal.pone.0139940 (PMC4598149; doi:10.1371/journal.pone.0139940)
Supplement: S1 Table — Sys systemic chemotherapy; EHD extrahepatic disease; OS overall survival; 5-FU Fluorouracil; Ox Oxaliplatin; Cis Cisplatin; Iri Irinotecan; Mit C Mitomycin C; LV Leucovorin; Pir Pirarubicin; Epi Epirubicin; UFT Tegafur-uracil; FUDR Floxuridine; Dox Doxorubicin. a Mean age (in years) bResponse = Complete Response + Partial Response. (DOCX) [file pone.0139940.s002.docx]

**Appendix Table 1.** Summary of Hepatic Arterial Infusion Articles Included

| Author | Pub year | N | Median age (years) | Patients pre-treated (%) | HAI drug | Sys  (%) | EHD  (%) | Grade 3-4 toxicities per patient | Termination due to technical complications (%) | Response rate^c^  (%) | Conversion to resectable (%) | Median OS (months) |
| --- | --- | --- | --- | --- | --- | --- | --- | --- | --- | --- | --- | --- |
| Sameshima S[1] | 2007 | 42 | 66^a^ | 0 | 5-FU | 0 | 0 | 0 | 7.1 | 57.1 |  | 29.1 |
| Kemeny N[2] | 2005 | 63 | 65 | 58.7 | FUDR, DEX, Mit C | 0 | 0 |  |  |  |  |  |
| *Pretreated* |  | 37 | 64 | 100 |  | 0 | 0 |  |  | 72.2 |  | 20.0 |
| *First-line* |  | 26 | 66 | 0 |  | 0 | 0 |  |  | 95.0 |  | 23.0 |
| Arai Y[3] | 2012 | 25 | 63 | 0 | 5-FU | 100 | 0 |  | 0 | 81.8 |  | 49.8 |
| Lee HJ[4] | 2011 | 14 | 64 | 100 | 5-FU, LV | 0 | 7.1 | 0.21 | 14.3 | 10.0 | 0 | 10.7 |
| Nishiofuku H | 2010 | 55 | 62 | 100 | 5-FU | 0 | 81.8 | 0.05 | 7.3 | 20.4 | 0 | 6.7 |
| Samaras P[5] | 2011 | 23 | 60 | 100 | FUDR, DEX | 52.2 | 17.4 | 1.00 | 0 | 26.1 | 34.8 | 15.6 |
| Chen Y[6] | 2010 | 32 | 59 ^a^ | 43.7 | OX, Iri, DOX | 0 | 0 | 1.25 |  | 46.9 |  | 17.7 |
| *First-line* |  | 18 |  | 0 |  |  | 0 |  |  |  |  | 22.4 |
| *Second-line* |  | 14 |  | 100 |  |  | 0 |  |  |  |  | 12.7 |
| Goéré D[7] | 2010 | 87 | 57 | 78.2 | OX | 100 | 2.3 |  | 8.0 |  | 16.1 |  |
| Khouri C[8] | 2010 | 17 | 62 | 100 | OX, RAL | 0 | 0 | 0.76 | 5.9 | 68.8 | 5.9 | 27.5 |
| Pilati P[9] | 2009 |  |  |  | FUDR, LV, DEX |  |  |  |  |  |  |  |
| *HAI alone* |  | 72 | 55 | 0 |  | 0 | 0 | 0.38 |  | 52.8 | 11.1 | 18.0 |
| *HAI + Sys* |  | 81 | 56 | 0 |  | 100 | 0 | 0.67 |  | 50.6 | 9.9 | 19.1 |
| Bouchahda M[10] | 2009 | 29 | 62 | 100 | OX, Iri, 5-FU | 0 | 31.0 | 0.41 | 34.5 | 34.5 | 13.8 | 18.0 |
| Fujimoto Y[11] | 2009 | 72 | 59 |  | 5-FU, Mit C | 0 | 100 |  |  | 37.5 | 9.7 | 18.0 |
| Seki H[12] | 2009 | 20 | 58 ^a^ |  | 5-FU | 0 | 20.0 | 0.05 | 0 | 85.0 | 5.0 | 30.1 |
| Idelevich E[13] | 2009 | 31 | 62 | 25.8 | FOLFIRI | 100 | 0 | 0.42 | 0 | 64.5 | 22.6 | 36.0 |
| Gallagher DJ[14] | 2007 | 39 | 58 | 100 | FUDR, DEX | 100 | 23.1 |  | 5.1 | 43.6 | 15.4 | 20.1 |
| Boige V[15] | 2008 | 44 | 56 | 100 | OX | 100 | 0 | 1.05 | 9.3 | 61.5 | 15.9 | 16.0 |
| Carnaghi C[16] | 2007 | 39 | 59 | 51.2 | 5-FU | 100 | 28.2 | 1.08 | 28.9 | 41.2 | 20.5 | 21.0 |
| *Pretreated* |  | 20 |  | 100 |  | 100 |  |  |  | 29.4 |  | 15.0 |
| *First-line* |  | 19 |  | 0 |  | 100 |  |  |  | 52.9 |  | 28.0 |
| *No EHD* |  | 28 |  |  |  |  | 0 |  |  | 50.0 |  | 26.0 |
| *EHD* |  | 11 |  |  |  |  | 100 |  |  | 20.0 |  | 17.0 |
| Pohlen U[17] | 2006 | 24 | 59 |  | 5-FU, LV | 0 | 0 | 0.29 | 0 | 50.0 |  | 14.0 |
| Tsutsumi S[18] | 2008 | 16 | 62 | 0 | 5-FU, LV | 100 | 0 | 0 |  | 87.5 |  | 22.0 |
| Del Freo A[19] | 2006 | 21 | 63 | 100 | 5-FU, OX | 0 | 0 | 0.48 |  | 23.8 | 9.5 | 10.6 |
| Kemeny NE[20] | 2006 | 62 | 57 | 0 | FUDR, LV | 0 | 0 |  | 4.8 | 47.5 |  | 24.4 |
| Ishibashi K[21] | 2005 | 20 | 62 | 0 | 5-FU, LV | 0 |  | 0.30 |  | 35.0 |  | 24.5 |
| Ducreux M[22] | 2005 | 28 | 60 | 75.0 | Ox | 100 | 0 | 0.85 | 38.5 | 85.7 | 18.5 | 27.0 |
| Ammori JB[23] | 2012 |  |  |  | - |  |  |  |  |  |  |  |
| *EHD* |  | 145 | 53 | 85.6 |  | 21.0 | 100 |  |  |  | 12.4 | 16.0 |
| *EHD & first-line* |  |  |  |  |  |  | 100 |  |  |  |  | 31.0 |
| *EHD & pretreated* |  |  |  |  |  |  | 100 |  |  |  |  | 14.0 |
| *No EHD* |  | 228 | 58 | 75.4 |  | 21.0 | 0 |  |  |  | 35.5 | 32.0 |
| *No EHD & first-line* |  |  |  | 0 |  |  | 0 |  |  |  |  | 49.0 |
| *No EHD & pretreated* |  |  |  | 100 |  |  | 0 |  |  |  |  | 27.0 |
| Qin B[24] | 2006 | 17 | 68 | 41.7 | 5-FU, Cis, LV | 0 | 0 | 0.35 | 0 | 52.9 | 5.9 | 26.0 |
| Van Riel JM[25] | 2004 | 25 | 61 | 100 | Iri | 0 | 44.0 | 0.76 | 8.0 | 13.6 |  | 8.1 |
| Zelek L[26] | 2003 | 31 | 54 | 0 | Pir | 100 | 6.5 |  |  | 48.4 | 35.5 | 20.5 |
| Naredi P[27] | 2003 | 21 | 68 |  | 5-FU, LV | 0 | 38.1 |  |  |  |  | 18.0 |
| Fallik D[28] | 2003 | 75 | 61 | 0 | Pir | 100 | 0 | 0.54 |  | 31.9 |  | 19.0 |
| Fazio N[29] | 2003 | 45 | 61 | 100 | 5-FU, Mit C, Iri | 0 | 44.4 |  |  | 36.4 |  | 11.7 |
| Mancuso A[30] | 2003 | 17 | 63 | 76.5 | OX | 0 | 0 | 0.41 |  | 46.7 | 5.9 | 19.0 |
| Neyns B[31] | 2006 | 9 | 56 | 100 | 5-FU, OX | 0 | 22.2 | 2.56 | 40.0 | 55.6 | 0 | 18.3 |
| Kemeny NE[2] | 2005 | 15 | 58 | 100 | FUDR | 100 | 0 |  |  | 86.7 | 0 | 22.0 |
| Kemeny NE[32] | 2009 | 49 | 59 | 53.1 | FUDR, DEX | 100 | 0 |  |  | 91.8 | 46.9 | 39.8 |
| *First-line* |  | 23 |  | 0 |  |  | 0 |  |  |  | 56.5 | 50.8 |
| *Pretreated* |  | 26 |  | 100 |  |  | 0 |  |  |  | 38.5 | 35.0 |
| Shimonov M[33] | 2005 | 15 | 65 ^a^ | 66.7 | UFT | 100 | 0 | 0 | 0 | 40.0 | 0 |  |
| Fiorentini G[34] | 2004 | 12 | 57 | 100 | OX | 0 | 0 |  | 0 | 33.3 | 0 | 13.0 |
| Mukai M[35] | 2006 | 15 |  | 0 | 5-FU, LV | 100 |  | 0.33 |  | 40.0 |  |  |
| Allen PJ[36] | 2005 | 544 | 60 ^a^ |  |  | 0 |  |  | 9.9 |  |  | 24.0 |
| Hosokawa A[37] | 2003 | 26 | 56 | 0 | 5-FU | 0 | 0 | 0.15 | 26.9 | 46.2 | 3.8 | 19.4 |
| Kerr DJ[38] | 2003 | 145 | 63 | 0 | 5-FU | 0 | 0 |  | 13.1 | 28.8 |  | 14.7 |
| Fiorentini G[39] | 2006 | 82 |  |  | FUDR |  |  |  |  |  |  |  |
| *HAI + Sys* |  | 42 |  | 0 |  | 100 | 0 |  | 4.8 | 47.5 |  | 20.0 |
| *HAI alone* |  | 36 |  | 0 |  | 0 | 0 |  | 0 | 41.7 |  | 14.0 |
| Tsimberidou AM[40] | 2013 | 58 |  |  |  |  |  |  | 7.9 |  |  |  |
| *KRAS (+)/unknown* |  | 28 | 62 |  | 5-FU, OX | 0 |  |  |  | 9.1 |  | 6.8 |
| *KRAS (-)* |  | 30 | 60 | 100 | OX | 100 |  |  |  | 23.1 |  | 11.5 |
| Melichar B[41] | 2012 | 22 | 66 ^a^ | 77.2 | FOLFOX | 9.1 |  | 0.23 | 22.7 | 21.1 | 9.1 | 11.0 |
| *First-line* |  | 5 |  | 0 |  |  |  |  |  | 50.0 | 40.0 | 6.0 |
| *Pretreated* |  | 17 |  | 100 |  |  |  |  |  | 13.3 | 0 | 11.0 |
| Chen Y[42] | 2012 | 31 | 60 a | 0 | UFT, OX, FUDR | 100 | 0 | 1.29 | 0 | 61.3 | 0 | 24.8 |
| Melichar B[43] | 2006 | 109 | 63 | 14.7 | 5-FU | 0 | 8.3 |  |  |  | 8.3 | 22.0 |
| *First-line* |  | 93 |  | 0 |  | 0 |  |  |  |  |  | 23.0 |
| *Second-line* |  | 16 |  | 100 |  | 0 |  |  |  |  |  | 14.0 |
| Neyns B[44] | 2008 | 8 | 60 | 100 | 5-FU, LV, OX (5/8) | 100 | 37.5 | 2.75 | 25.0 | 62.5 |  |  |
| Kim JC[45] | 2006 | 29 | 53 ^a^ |  | 5-FU, Mit C | 100 | 0 | 0.21 |  | 31.0 |  | 38.0 |
| Melichar B[46] | 2012 | 60 | 61 | 47.1 | 5-FU, LV, Iri | 0 |  |  |  | 39.2 | 11.7 | 23.0 |
| Seki H[47] | 2008 | 135 | 62^a^ | 32.6 | 5-FU | 0 | 34.8 |  | 22.2 |  | 5.9 |  |
| *Conventional catheter* |  | 10 |  | 20.0 |  | 0 | 10.0 |  | 80.0 | 50.0 |  | 13.1 |
| *Side-hole original fixed catheter tip* |  | 77 |  | 28.6 |  | 0 | 31.2 |  | 19.5 | 63.6 |  | 21.1 |
| *Side-hole modified fixed catheter tip* |  | 24 |  | 29.2 |  | 0 | 45.8 |  | 16.7 | 79.2 |  | 22.5 |
| *Side-hole long tapered catheter placement* |  | 24 |  | 54.2 |  | 0 | 45.8 |  | 12.5 | 70.8 |  | 23.1 |
| Libra M[48] | 2004 | 20 | 59 |  | FUDR | 0 |  |  |  | 30.0 |  | 17.0 |
| Fiorentini G[49] | 2004 | 42 |  | 0 | 5-FU, Mit C, Epi | 0 | 0 |  |  |  | 0 |  |
| *High doses + chemofiltration* |  | 20 |  | 0 |  | 0 | 0 |  |  |  | 0 | 17.0 |
| *Prolonged low doses* |  | 22 |  | 0 |  | 0 | 0 |  |  |  | 0 | 11.0 |
| Milandri M[50] | 2005 | 14 | 62 | 64.3 | 5-FU, Mit C | 0 |  |  | 14.3 | 14.3 | 7.1 | 15.0 |

Sys systemic chemotherapy; EHD extrahepatic disease; OS overall survival; 5-FU Fluorouracil; Ox Oxaliplatin; Cis Cisplatin; Iri Irinotecan; Mit C Mitomycin C; LV Leucovorin; Pir Pirarubicin; Epi Epirubicin; UFT Tegafur-uracil; FUDR Floxuridine; Dox Doxorubicin

^a^ Mean age (in years)

^b^Response = Complete Response + Partial Response

References

1. Sameshima S, Horikoshi H, Motegi K, Tomozawa S, Hirayama I, Saito T, et al. Outcomes of hepatic artery infusion therapy for hepatic metastases from colorectal carcinoma after radiological placement of infusion catheters. . 2007;33: 741-745.

2. Kemeny N, Eid A, Stockman J, Gonen M, Schwartz L, Tetzlaff E, et al. Hepatic arterial infusion of floxuridine and dexamethasone plus high-dose Mitomycin C for patients with unresectable hepatic metastases from colorectal carcinoma. J Surg Oncol. 2005;91: 97-101.

3. Arai Y, Ohtsu A, Sato Y, Aramaki T, Kato K, Hamada M, et al. Phase I/II Study of Radiologic Hepatic Arterial Infusion of Fluorouracil Plus Systemic Irinotecan for Unresectable Hepatic Metastases from Colorectal Cancer: Japan Clinical Oncology Group Trial 0208-DI. . 2012;23: 1261-1267.

4. Lee HJ, Lee YS, Lee K, Kim SY, Yoon CJ, Shin D, et al. Efficacy and Safety of Hepatic Arterial Infusion of Fluorouracil with Leucovorin as Salvage Treatment for Refractory Liver Metastases from Colorectal Cancer. Korean J Intern Med. 2011;26: 82-88.

5. Samaras P, Breitenstein S, Haile SR, Stenner-Liewen F, Heinrich S, Feilchenfeldt J, et al. Selective intra-arterial chemotherapy with floxuridine as second- or third-line approach in patients with unresectable colorectal liver metastases. . 2011;18: 1924-1931.

6. Chen Y, Yan Z, Wang J, Wang X, Luo J, Liu Q. Hepatic arterial infusion with oxaliplatin, irinotecan and doxifluridine for unresectable liver metastases of colorectal cancer. Anticancer Res. 2010;30: 3045-3049.

7. Goéré D, Deshaies I, De Baere T, Boige V, Malka D, Dumont F, et al. Prolonged survival of initially unresectable hepatic colorectal cancer patients treated with hepatic arterial infusion of oxaliplatin followed by radical surgery of metastases. Ann Surg. 2010;251: 686-691.

8. Khouri C, Guiu B, Cercueil JP, Chauffert B, Ladoire S, Ghiringhelli F. Raltitrexed and oxaliplatin hepatic arterial infusion for advanced colorectal cancer: A retrospective study. Anticancer Drugs. 2010;21: 656-661.

9. Pilati P, Mammano E, Mocellin S, Tessari E, Lise M, Nitti D. Hepatic arterial infusion for unresectable colorectal liver metastases combined or not with systemic chemotherapy. Anticancer Res. 2009;29: 4139-4144.

10. Bouchahda M, Adam R, Giacchetti S, Castaing D, Brezault-Bonnet C, Hauteville D, et al. Rescue chemotherapy using multidrug chronomodulated hepatic arterial infusion for patients with heavily pretreated metastatic colorectal cancer. Cancer. 2009;115: 4990-4999.

11. Fujimoto Y, Akasu T, Yamamoto S, Fujita S, Moriya Y. Long-term results of hepatectomy after hepatic arterial infusion chemotherapy for initially unresectable hepatic colorectal metastases. . 2009;13: 1643-1650.

12. Seki H, Ozaki T, Shiina M. Hepatic arterial infusion chemotherapy using fluorouracil followed by systemic therapy using oxaliplatin plus fluorouracil and leucovorin for patients with unresectable liver metastases from colorectal cancer. Cardiovasc Intervent Radiol. 2009;32: 679-686.

13. Idelevich E, Greif F, Mavor E, Miller R, Kashtan H, Susmalian S, et al. Phase II study of UFT with leucovorin plus hepatic arterial infusion with irinotecan, 5-fluorouracil and leucovorin for non-resectable liver metastases of colorectal cancer. Chemotherapy. 2009;55: 76-82.

14. Gallagher DJ, Capanu M, Raggio G, Kemeny N. Hepatic arterial infusion plus systemic irinotecan in patients with unresectable hepatic metastases from colorectal cancer previously treated with systemic oxaliplatin: A retrospective analysis. . 2007;18: 1995-1999.

15. Boige V, Malka D, Elias D, Castaing M, De Baere T, Goere D, et al. Hepatic arterial infusion of oxaliplatin and intravenous LV5FU2 in unresectable liver metastases from colorectal cancer after systemic chemotherapy failure. . 2008;15: 219-226.

16. Carnaghi C, Santoro A, Rimassa L, Doci R, Rosati R, Pedicini V, et al. The efficacy of hybrid chemotherapy with intravenous oxaliplatin and folinic acid and intra-hepatic infusion of 5-fluorouracil in patients with colorectal liver metastases: A phase II study. Invest New Drugs. 2007;25: 479-485.

17. POHLEN U, RIEGER H, MANSMANN U, BERGER G, BUHR HJ. Hepatic Arterial Infusion (HAI). Comparison of 5-Fluorouracil, Folinic Acid, Interferon Alpha-2b and Degradable Starch Microspheres versus 5-Fluorouracil and Folinic Acid in Patients with Non-resectable Colorectal Liver Metastases. Anticancer Research. 2006;26: 3957-3964.

18. Tsutsumi S, Yamaguchi S, Tsuboi K, Fukasawa T, Tabe Y, Asao T, et al. Hepaticarterialinfusioncombined with oralUFT/UZELsystemicchemotherapy for unresectablelivermetastasis of colorectalcancer. . 2008;55: 1419.

19. Del Freo A, Fiorentini G, Sanguinetti F, Muttini MP, Pennucci C, Mambrini A, et al. Hepatic arterial chemotherapy with oxaliplatin, folinic acid and 5-fluorouracil in pre-treated patients with liver metastases from colorectal cancer. In Vivo. 2006;20: 743-746.

20. Kemeny NE, Niedzwiecki D, Hollis DR, Lenz H-, Warren RS, Naughton MJ, et al. Hepatic arterial infusion versus systemic therapy for hepatic metastases from colorectal cancer: A randomized trial of efficacy, quality of life, and molecular markers (CALGB 9481). . 2006;24: 1395-1403.

21. Ishibashi K, Yoshimatsu K, Yokomizo H, Umehara A, Yoshida K, Fujimoto T, et al. Low-dose leucovorin and 5-fluorouracil for unresectable multiple liver metastasis from colorectal cancer. Anticancer Res. 2005;25: 4747-4752.

22. Ducreux M, Ychou M, Laplanche A, Gamelin E, Lasser P, Husseini F, et al. Hepatic arterial oxaliplatin infusion plus intravenous chemotherapy in colorectal cancer with inoperable hepatic metastases: A trial of the Gastrointestinal Group of the Fédération Nationale des Centres de Lutte Contre le Cancer. . 2005;23: 4881-4887.

23. Ammori JB, D'Angelica MI, Fong Y, Cercek A, Dematteo RP, Allen PJ, et al. Hepatic artery infusional chemotherapy in patients with unresectable colorectal liver metastases and extrahepatic disease. J Surg Oncol. 2012;106: 953-958.

24. Qin B, Kato K, Mitsugi K, Nakamura M, Tanaka R, Baba E, et al. Feasibility study of ambulatory continuous infusion of 5-fluorouracil followed by cisplatin through hepatic artery for metastatic colorectal cancer. . 2006;57: 114-119.

25. Van Riel J, Van Groeningen C, De Greve J, Gruia G, Pinedo H, Giaccone G. Continuous infusion of hepatic arterial irinotecan in pretreated patients with colorectal cancer metastatic to the liver. . 2004;15: 59-63.

26. Zelek L, Bugat R, Cherqui D, Ganem G, Valleur P, Guimbaud R, et al. Multimodal therapy with intravenous biweekly leucovorin, 5-fluorouracil and irinotecan combined with hepatic arterial infusion pirarubicin in non-resectable hepatic metastases from colorectal cancer (a European Association for Research in Oncology Trial). . 2003;14: 1537-1542.

27. Naredi P, Öman M, Blind P-, Lindnér P, Gustavsson B, Hafström L. A comparison between hepatic artery ligation and portal 5-Fu infusion versus 5-Fu intra arterial infusion for colorectal liver metastases. . 2003;29: 459-466.

28. Fallik D, Ychou M, Jacob J, Colin P, Seitz JF, Baulieux J, et al. Hepatic arterial infusion using pirarubicin combined with systemic chemotherapy: A phase II study in patients with nonresectable liver metastases from colorectal cancer. . 2003;14: 856-863.

29. Fazio N, Orsi F, Grasso RF, Ferretyi G, Medici M, Rocca A, et al. Hepatic Intra-Arterial Chemotherapy using a Percutaneous Catheter in Pretreated Patients with Metastatic Colorectal Carcinoma. Anticancer Res. 2003;23: 5023-5030.

30. Mancuso A, Giuliani R, Accettura C, Palma M, D'Auria G, Cecere F, et al. Hepatic arterial continuous infusion (HACI) of oxaliplatin in patients with unresectable liver metastases from colorectal cancer. Anticancer Res. 2003;23: 1917-1922.

31. Neyns B, Van Nieuwenhove Y, Aerts M, Fontaine C, Vermeij J, Schallier D, et al. Hepatic arterial infusion of oxaliplatin and L-folinic acid-modulated 5-fluorouracil for colorectal cancer liver metastases. Anticancer Res. 2006;26: 611-619.

32. Kemeny NE, Huitzil Melendez FD, Capanu M, Paty PB, Fong Y, Schwartz LH, et al. Conversion to resectability using hepatic artery infusion plus systemic chemotherapy for the treatment of unresectable liver metastases from colorectal carcinoma. . 2009;27: 3465-3471.

33. Shimonov M, Hayat H, Chaitchik S, Brener J, Schachter P, Czerniak A. Combined systemic chronotherapy and hepatic artery infusion for the treatment of metastatic colorectal cancer confined to the liver: A pilot study. Chemotherapy. 2005;51: 111-115.

34. Fiorentini G, Rossi S, Dentico P, Meucci F, Bonechi F, Bernardeschi P, et al. Oxaliplatin hepatic arterial infusion chemotherapy for hepatic metastases from colorectal cancer: A phase I-II clinical study. Anticancer Res. 2004;24: 2093-2096.

35. Mukai M, Oida Y, Tajima T, Kishima K, Ninomiya H, Sato S, et al. Alternating hepatic arterial infusion and systemic chemotherapy for stage IV colorectal cancer with synchronous liver metastasis. Oncol Rep. 2006;16: 865-870.

36. Allen PJ, Nissan A, Picon AI, Kemeny N, Dudrick P, Ben-Porat L, et al. Technical complications and durability of hepatic artery infusion pumps for unresectable colorectal liver metastases: An institutional experience of 544 consecutive cases. J Am Coll Surg. 2005;201: 57-65.

37. Hosokawa A, Yamada Y, Shimada Y, Muro K, Matsumura Y, Fujita S, et al. Weekly hepatic arterial infusion of 5-fluorouracil and subsequent systemic chemotherapy for liver metastases from colorectal cancer. Jpn J Clin Oncol. 2003;33: 132-135.

38. Kerr DJ, McArdle CS, Ledermann J, Taylor I, Sherlock DJ, Schlag PM, et al. Intrahepatic arterial versus intravenous fluorouracil and folinic acid for colorectal cancer liver metastases: A multicentre randomised trial. Lancet. 2003;361: 368-373.

39. Fiorentini G, Cantore M, Rossi S, Vaira M, Tumolo S, Dentico P, et al. Hepatic arterial chemotherapy in combination with systemic chemotherapy compared with hepatic arterial chemotherapy alone for liver metastases from colorectal cancer: Results of a multi-centric randomized study. In Vivo. 2006;20: 707-710.

40. Tsimberidou AM, Leick MB, Lim J, Fu S, Wheler J, Piha-Paul SA, et al. Dose-finding study of hepatic arterial infusion of oxaliplatin-based treatment in patients with advanced solid tumors metastatic to the liver. Cancer Chemother Pharmacol. 2013;71: 389-397.

41. Melichar B, Ferko A, Krajina A, Rousková L, Dvorák J, Svébisova H, et al. Hepatic arterial infusion of oxaliplatin, 5-fluorouracil and leucovorin in patients with liver metastases from colorectal carcinoma. . 2012;17: 677-683.

42. Chen Y, Wang X, Yan Z, Wang J, Luo J, Liu Q. Hepatic arterial infusion with irinotecan, oxaliplatin, and floxuridine plus systemic chemotherapy as first-line treatment of unresectable liver metastases from colorectal cancer. Onkologie. 2012;35: 480-484.

43. Melichar B, Voboril Z, Cerman Jr. J, Melicharová K, Mergancová J, Voboril R, et al. Survival of patients with colorectal cancer liver metastases treated by regional chemotherapy. Hepatogastroenterology. 2006;53: 426-434.

44. Neyns B, Aerts M, Van Nieuwenhove Y, Fontaine C, De Coster L, Schallier D, et al. Cetuximab with hepatic arterial infusion of chemotherapy for the treatment of colorectal cancer liver metastases. Anticancer Res. 2008;28: 2459-2467.

45. Kim JC, Kim HC, Lee KH, Yu CS, Kim TW, Chang HM, et al. Hepatic arterial infusion alternating with systemic chemotherapy in patients with non-resectable hepatic metastases from colorectal cancer. J Gastroenterol Hepatol. 2006;21: 1026-1035.

46. Melichar B, Voboril Z, Krajina A, Malírová E, Weiner P, Nová M, et al. Hepatic arterial infusion of irinotecan, 5-fluorouracil and leucovorin in patients with liver metastases from colorectal carcinoma. Anticancer Res. 2012;32: 5487-5494.

47. Seki H, Ozaki T, Shiina M. Side-hole catheter placement for hepatic arterial infusion chemotherapy in patients with liver metastases from colorectal cancer: Long-term treatment and survival benefit. Am J Roentgenol. 2008;190: 111-120.

48. Libra M, Navolanic PM, Talamini R, Cecchin E, Sartor F, Tumolo S, et al. Thymidylate synthetase mRNA levels are increased in liver metastases of colorectal cancer patients resistant to fluoropyrimidine-based chemotherapy. BMC Cancer. 2004;4.

49. Fiorentini G, Poddie DB, Cantore M, Rossi S, Tumolo S, Dentico P, et al. Hepatic intra-arterial chemotherapy (HIAC) of high dose mitomycin and epirubicin combined with caval chemofiltration versus prolonged low doses in liver metastases from colorectal cancer: A prospective randomized clinical study. . 2004;16: 51-54.

50. Milandri M, Calzolari F, Passardi A, Ridolfi R, Tison C, Giampalma E, et al. Intra-arterial chemotherapy for liver metastases from colorectal cancer. . 2005;4.
