# Supplementary material for: Comparative Effectiveness of Hepatic Artery Based Therapies for Unresectable Colorectal Liver Metastases: A Meta-Analysis
Source: PLoS One. 2015 Oct 8;10(10):e0139940. doi: 10.1371/journal.pone.0139940 (PMC4598149; doi:10.1371/journal.pone.0139940)
Supplement: S2 Table — Sys systemic chemotherapy; EHD extrahepatic disease; OS overall survival. a Mean age (in years) bSubsequent HAI cResponse = Complete Response + Partial Response. (DOCX) [file pone.0139940.s003.docx]

**Appendix Table 2.** Summary of Radioembolization Articles Included

| Author | Pub  Year | N | Median age (years) | Patients pre- treated (%) | Resin microsphere used – Yes/No | Sys  (%) | EHD  (%) | Grade 3-4 toxicities per patient | Response rate^c^ (%) | Conversion to resectable (%) | Median OS (months) |
| --- | --- | --- | --- | --- | --- | --- | --- | --- | --- | --- | --- |
| Martin LK[1] | 2012 | 24 | 63 | 100 | - | 0 | 54.2 |  |  | 0 | 8.9 |
| Bester L[2] | 2012 | 224 | 67 | 100 | Yes | 0 | 37.9 |  |  |  | 11.9 |
| Cosimelli M[3] | 2010 | 50 | 67 | 100 | Yes | 0 | 22.0 | 0 | 24.0 |  | 12.6 |
| Hong K[4] | 2009 | 15 | 64 | 100 |  | 0 | 33.3 |  |  |  | 6.9 |
| Kosmider S[5] | 2011 | 19 | 62 | 0 | Yes | 100 | 26.3 | 1.21 | 89.5 |  | 29.4 |
| *No EHD* |  | 12 |  | 0 | Yes | 100 | 0 |  |  |  | 37.8 |
| *EHD* |  | 5 |  | 0 | Yes | 100 | 100 |  |  |  | 13.4 |
| Hendlisz A[6] | 2010 | 21 | 62 | 100 | Yes | 100 | 0 | 0.05 | 9.5 | 4.8 | 10.0 |
| Van Hazel GA[7] | 2009 | 25 | 59 | 100 | Yes | 100 | 48.0 | 0.48 | 47.8 |  | 12.2 |
| Mulcahy MF[8] | 2009 | 72 | 61 | 94.4 |  | 0 | 40.3 | 0.26 | 40.6 |  | 14.5 |
| *No EHD* |  | 43 |  |  |  | 0 | 0 |  |  |  | 21.0 |
| *EHD* |  | 29 |  |  |  | 0 | 100 |  |  |  | 7.9 |
| Stubbs RS[9] | 2006 | 100 | 61 |  | Yes | 80·0 | 25.0 |  |  |  | 11.0 |
| *Subsequent HAI* |  | 80 |  |  |  | 100^b^ |  |  |  |  | 12.6 |
| *No HAI* |  | 20 |  |  |  | 0 |  |  |  |  | 2.6 |
| Sharma RA[10] | 2007 | 20 | 59 | 0 | Yes | 100 | 65.0 |  | 90.0 | 10.0 |  |
| Kennedy A[11] | 2006 | 208 | 62 | 100 | Yes | 0 |  | 0.22 | 35.6 |  |  |
| Lewandowski RJ[12] | 2005 | 27 | 68 | 88.9 |  | 0 | 77.8 |  |  |  | 9.4 |
| Murthy R[13] | 2005 | 12 | 58 | 100 | Yes | 16·7 | 83.3 | 0.08 | 0 | 0 | 4.5 |
| Van Hazel G[14] | 2004 | 11 |  | 0 | Yes | 100 | 18.2 | 1.18 | 90.9 |  | 29.4 |
| Seidensticker R[15] | 2012 | 29 | 62 ^a^ | 100 | Yes | 31·0 | 48.3 | 0.10 | 42.9 | 0 | 8.3 |
| Cianni R[16] | 2009 | 41 | 61 ^a^ | 100 | Yes | 0 | 9.8 |  | 46.3 |  | 11.6 |
| Jakobs TF[17] | 2008 | 41 | 61 ^a^ | 100 | Yes | 0 | 17.1 |  | 19.4 |  | 10.5 |
| Sato KT[18] | 2008 | 51 | 63 ^a^ | 100 |  | 0 | 49.6 |  |  |  | 15.2 |
| Chua TC[19] | 2011 | 140 | 64 | 94·3 | Yes | 34·3 | 36.4 |  | 32.1 | 0.7 | 9.0 |
| *RE alone* |  | 92 |  |  |  | 0 |  |  |  |  | 7.0 |
| *RE + Sys* |  | 48 |  |  |  | 100 |  |  |  |  | 13.0 |
| Omed A[20] | 2010 | 7 | 56 | 100 |  | 0 |  |  |  |  | 7.5 |
| Stuart JE[21] | 2008 | 13 | 58 | 100 | Yes | 23·1 | 38.5 | 0.03 |  |  | 11.7 |
| Mancini R[22] | 2006 | 35 |  | 100 | Yes | 0 |  |  | 12.5 |  |  |
| Lim L[23] | 2005 | 32 | 64 | 87.5 | Yes | 12·5 | 15.6 |  | 32.3 |  |  |
| Nace GW[24] | 2011 | 51 | 64 | 100 | Yes | 66·7 | 54.9 |  | 12.9 |  | 10.2 |
| *RE + Sys* |  | 17 |  | 100 | Yes | 100 |  |  |  |  | 17.0 |
| *RE alone* |  | 34 |  | 100 | Yes | 0 |  |  |  |  | 8.2 |
| *EHD* |  | 28 |  | 100 |  |  | 100 |  |  |  | 6.7 |
| *No EHD* |  | 20 |  | 100 |  |  | 0 |  |  |  | 17.0 |

Sys systemic chemotherapy; EHD extrahepatic disease; OS overall survival

^a^ Mean age (in years)

^b^Subsequent HAI

^c^Response = Complete Response + Partial Response

References

1. Martin LK, Cucci A, Wei L, Rose J, Blazer M, Schmidt C, et al. Yttrium-90 radioembolization as salvage therapy for colorectal cancer with liver metastases. . 2012;11: 195-199.

2. Bester L, Meteling B, Pocock N, Pavlakis N, Chua TC, Saxena A, et al. Radioembolization versus standard care of hepatic metastases: Comparative retrospective cohort study of survival outcomes and adverse events in salvage patients. . 2012;23: 96-105.

3. Cosimelli M, Golfieri R, Cagol PP, Carpanese L, Sciuto R, Maini CL, et al. Multi-centre phase II clinical trial of yttrium-90 resin microspheres alone in unresectable, chemotherapy refractory colorectal liver metastases. Br J Cancer. 2010;103: 324-331.

4. Hong K, McBride JD, Georgiades CS, Reyes DK, Herman JM, Kamel IR, et al. Salvage Therapy for Liver-dominant Colorectal Metastatic Adenocarcinoma: Comparison between Transcatheter Arterial Chemoembolization versus Yttrium-90 Radioembolization. . 2009;20: 360-367.

5. Kosmider S, Tan TH, Yip D, Dowling R, Lichtenstein M, Gibbs P. Radioembolization in combination with systemic chemotherapy as first-line therapy for liver metastases from colorectal cancer. . 2011;22: 780-786.

6. Hendlisz A, Van Den Eynde M, Peeters M, Maleux G, Lambert B, Vannoote J, et al. Phase III trial comparing protracted intravenous fluorouracil infusion alone or with yttrium-90 resin microspheres radioembolization for liver-limited metastatic colorectal cancer refractory to standard chemotherapy. . 2010;28: 3687-3694.

7. Van Hazel GA, Pavlakis N, Goldstein D, Olver IN, Tapner MJ, Price D, et al. Treatment of fluorouracil-refractory patients with liver metastases from colorectal cancer by using yttrium-90 resin microspheres plus concomitant systemic irinotecan chemotherapy. . 2009;27: 4089-4095.

8. Mulcahy MF, Lewandowski RJ, Ibrahim SM, Sato KT, Ryu RK, Atassi B, et al. Radioembolization of colorectal hepatic metastases using Yttrium-90 microspheres. Cancer. 2009;115: 1849-1858.

9. Stubbs RS, O'Brien I, Correia MM. Selective internal radiation therapy with 90Y microspheres for colorectal liver metastases: Single-centre experience with 100 patients. ANZ J Surg. 2006;76: 696-703.

10. Sharma RA, Van Hazel GA, Morgan B, Berry DP, Blanshard K, Price D, et al. Radioembolization of liver metastases from colorectal cancer using Yttrium-90 microspheres with concomitant systemic oxaliplatin, fluorouracil, and leucovorin chemotherapy. . 2007;25: 1099-1106.

11. Kennedy AS, Coldwell D, Nutting C, Murthy R, Wertman Jr. DE, Loehr SP, et al. Resin 90Y-microsphere brachytherapy for unresectable colorectal liver metastases: Modern USA experience. . 2006;65: 412-425.

12. Lewandowski RJ, Thurston KG, Goin JE, Wong CO, Gates VL, Buskirk MV, et al. 90Y Microsphere (TheraSphere) Treatment for Unresectable Colorectal Cancer Metastases of the Liver: Response to Treatment at Targeted Doses of 135–150 Gy as Measured by [18F]Fluorodeoxyglucose Positron Emission Tomography and Computed Tomographic Imaging. . 2005;16: 1641-1651.

13. Murthy R, Xiong H, Nunez R, Cohen AC, Barron B, Szklaruk J, et al. Yttrium 90 resin microspheres for the treatment of unresectable colorectal hepatic metastases after failure of multiple chemotherapy regimens: Preliminary results. . 2005;16: 937-945.

14. Van Hazel G, Blackwell A, Anderson J, Price D, Moroz P, Bower G, et al. Randomised phase 2 trial of SIR-Spheres® plus fluorouracil/leucovorin chemotherapy versus fluorouracil/leucovorin chemotherapy alone in advanced colorectal cancer. J Surg Oncol. 2004;88: 78-85.

15. Seidensticker R, Denecke T, Kraus P, Seidensticker M, Mohnike K, Fahlke J, et al. Matched-pair comparison of radioembolization plus best supportive care versus best supportive care alone for chemotherapy refractory liver-dominant colorectal metastases. Cardiovasc Intervent Radiol. 2012;35: 1066-1073.

16. Cianni R, Urigo C, Notarianni E, Saltarelli A, Salvatori R, Pasqualini V, et al. Selective internal radiation therapy with SIR-spheres for the treatment of unresectable colorectal hepatic metastases. Cardiovasc Intervent Radiol. 2009;32: 1179-1186.

17. Jakobs TF, Hoffmann R-, Dehm K, Trumm C, Stemmler H-, Tatsch K, et al. Hepatic Yttrium-90 Radioembolization of Chemotherapy-refractory Colorectal Cancer Liver Metastases. . 2008;19: 1187-1195.

18. Sato KT, Lewandowski RJ, Mulcahy MF, Atassi B, Ryu RK, Gates VL, et al. Unresectable chemorefractory liver metastases: Radioembolization with 90Y microspheres - Safety, efficacy, and survival. Radiology. 2008;247: 507-515.

19. Chua TC, Bester L, Saxena A, Morris DL. Radioembolization and systemic chemotherapy improves response and survival for unresectable colorectal liver metastases. J Cancer Res Clin Oncol. 2011;137: 865-873.

20. Omed A, Lawrance JAL, Murphy G, Laasch H-, Wilson G, Illidge T, et al. A retrospective analysis of selective internal radiation therapy (SIRT) with yttrium-90 microspheres in patients with unresectable hepatic malignancies. Clin Radiol. 2010;65: 720-728.

21. Stuart JE, Tan B, Myerson RJ, Garcia-Ramirez J, Goddu SM, Pilgram TK, et al. Salvage Radioembolization of Liver-dominant Metastases with a Resin-based Microsphere: Initial Outcomes. . 2008;19: 1427-1433.

22. Mancini R, Carpanese L, Sciuto R, Pizzi G, Golfieri R, Giampalma L, et al. A multicentric phase II clinical trial on intra-arterial hepatic radiotherapy with 90Yttrium SIR-spheres in unresectable, colorectal liver metastases refractory to i.v. chemotherapy: Preliminary results on toxicity and response rates. In Vivo. 2006;20: 711-714.

23. Lim L, Gibbs P, Yip D, Shapiro JD, Dowling R, Smith D, et al. Prospective study of treatment with selective internal radiation therapy spheres in patients with unresectable primary or secondary hepatic malignancies. Intern Med J. 2005;35: 222-227.

24. Nace G, Steel J, Amesur N, Zajko A, Nastasi B, Joyce J, et al. Yttrium-90 radioembolization for colorectal cancer liver metastases: a single institution experience. . 2011;2011:571261.
