# Supplementary material for: Comparative Effectiveness of Hepatic Artery Based Therapies for Unresectable Colorectal Liver Metastases: A Meta-Analysis
Source: PLoS One. 2015 Oct 8;10(10):e0139940. doi: 10.1371/journal.pone.0139940 (PMC4598149; doi:10.1371/journal.pone.0139940)
Supplement: S3 Table — Sys systemic chemotherapy; EHD extrahepatic disease; OS overall survival; 5-FU Fluorouracil; Cis Cisplatin; Iri Irinotecan; Mit C Mitomycin C; Gem Gemcitabine; Dox Doxorubicin. a Mean age (in years) bResponse = Complete Response + Partial Response. (DOCX) [file pone.0139940.s004.docx]

**Appendix Table 3.** Summary of Transcatheter Arterial Chemoembolization Articles Included

| Author | Pub year | N | Median age | Patients pre-treated (%) | Type of TACE agent | Sys (%) | DEBIRI used (Y/N) | EHD(%) | Grade 3-4 toxicities per patient | Response rate^c^ (%) | Conversion to resectable (%) | Median OS (months) |
| --- | --- | --- | --- | --- | --- | --- | --- | --- | --- | --- | --- | --- |
| Hong K[1] | 2009 | 21 | 67 ^a^ | 100 | Cis + Dox + Mit C | 0 | N | 42.9 |  |  |  | 7.7 |
| Martin RC[2] | 2011 | 55 | 60 | 100 | Iri | 29.1 | Y | 49.1 | 0.09 | 67.9 |  | 19.0 |
| Martin RC[3] | 2009 | 55 | 62 | 98.2 | Iri | 0 | Y | 45.5 |  | 38.9 | 7.2 | 8.1 |
| Fiorentini G[4] | 2012 | 36 | 64 ^a^ | 100 | Iri | 0 | Y | 0 |  | 68.6 |  | 22.0 |
| Vogl TJ[5] | 2009 | 463 | 62 ^a^ | 100 |  | 0 | N | 0 |  | 14.7 |  | 14.0 |
| *Mit C only* |  | 243 |  | 100 | Mit C | 0 | N |  |  | 12.5 |  | 14.0 |
| *Mit C + Gem* |  | 153 |  | 100 | Mit C + Gem | 0 | N |  |  | 11.1 |  | 13.9 |
| *Mit C + Iri* |  | 67 |  | 100 | Mit C + Iri | 0 | N |  |  | 19.4 |  | 14.0 |
| You YT[6] | 2006 | 40 | 60 |  | 5-FU + Gem | 100 | N | 0 | 0.10 | 47.5 |  | 16.0 |
| Albert M[7] | 2011 | 121 | 62 ^a^ |  | Mit C + Dox + Cis | 0 | N | 46.3 | 0.19 | 2.1 | 0 | 9.0 |
| *No EHD* |  | 65 |  |  | Mit C + Dox + Cis | 0 |  | 0 |  |  |  | 11.0 |
| *EHD* |  | 56 |  |  | Mit C + Dox + Cis | 0 |  | 100 |  |  |  | 8.0 |
| Fiorentini G[8] | 2007 | 20 |  | 100 | Iri | 0 | Y |  | 0.25 | 80.0 |  |  |
| Aliberti C[9] | 2011 | 82 | 62 | 100 | Iri | 0 | Y | 0 |  |  | 2.4 | 25.0 |
| Narayanan G[10] | 2013 | 28 |  | 92.9 | Iri | 0 | Y | 21.4 |  | 45.0 |  | 13.3 |
| Martin RC[11] | 2009 | 30 | 55 |  | Iri | 0 | Y | 53.3 | 0.10 | 75.0 |  |  |
| Martin RC[12] | 2012 | 10 | 63 | 0 | Iri | 100 | Y | 50.0 | 0.60 | 90.0 | 40.0 | 15.2 |
| Eichler K[13] | 2012 | 11 | 64 ^a^ | 72.7 | Iri | 0 | Y | 0 |  | 25.0 |  |  |

Sys systemic chemotherapy; EHD extrahepatic disease; OS overall survival; 5-FU Fluorouracil; Cis Cisplatin; Iri Irinotecan; Mit C Mitomycin C; Gem Gemcitabine; Dox Doxorubicin

^a^ Mean age (in years)

^b^Response = Complete Response + Partial Response

References

1. Hong K, McBride JD, Georgiades CS, Reyes DK, Herman JM, Kamel IR, et al. Salvage Therapy for Liver-dominant Colorectal Metastatic Adenocarcinoma: Comparison between Transcatheter Arterial Chemoembolization versus Yttrium-90 Radioembolization. . 2009;20: 360-367.

2. Martin RCG, Joshi J, Robbins K, Tomalty D, Bosnjakovik P, Derner M, et al. Hepatic intra-arterial injection of drug-eluting bead, irinotecan (DEBIRI) in unresectable colorectal liver metastases refractory to systemic chemotherapy: Results of multi-institutional study. . 2011;18: 192-198.

3. Martin RCG, Robbins K, Tomalty D, O'Hara R, Bosnjakovic P, Padr R, et al. Transarterial chemoembolisation (TACE) using irinotecan-loaded beads for the treatment of unresectable metastases to the liver in patients with colorectal cancer: An interim report. . 2009;7: 80.

4. Fiorentini G, Aliberti C, Tilli M, Mulazzani L, Graziano F, Giordani P, et al. Intra-arterial infusion of irinotecan-loaded drug-eluting beads (DEBIRI) versus intravenous therapy (FOLFIRI) for hepatic metastases from colorectal cancer: Final results of a phase III study. Anticancer Res. 2012;32: 1387-1395.

5. Vogl TJ, Gruber T, Balzer JO, Eichler K, Hammerstingl R, Zangos S. Repeated transarterial chemoembolization in the treatment of liver metastases of colorectal cancer: Prospective study. Radiology. 2009;250: 281-289.

6. You Y-, Changchien C-, Huang J-, Ng K-. Combining systemic chemotherapy with chemoembolization in the treatment of unresectable hepatic metastases from colorectal cancer. Int J Colorectal Dis. 2006;21: 33-37.

7. Albert M, Kiefer MV, Sun W, Haller D, Fraker DL, Tuite CM, et al. Chemoembolization of colorectal liver metastases with cisplatin, doxorubicin, mitomycin C, ethiodol, and polyvinyl alcohol. Cancer. 2011;117: 343-352.

8. Fiorentini G, Aliberti C, Turrisi G, Del Conte A, Rossi S, Benea G, et al. Intraarterial hepatic chemoembolization of liver metastases from colorectal cancer adopting irinotecan-eluting beads: Results of a phase II clinical study. In Vivo. 2007;21: 1085-1092.

9. Aliberti C, Fiorentini G, Muzzio PC, Pomerri F, Tilli M, Dallara S, et al. Trans-arterial chemoembolization of metastatic colorectal carcinoma to the liver adopting DC Bead®, drug-eluting bead loaded with irinotecan: Results of a phase II clinical study. Anticancer Res. 2011;31: 4581-4587.

10. Narayanan G, Barbery K, Suthar R, Guerrero G, Arora G. Transarterial chemoembolization using DEBIRI for treatment of hepatic metastases from colorectal cancer. Anticancer Res. 2013;33: 2077-2083.

11. Martin RCG, Joshi J, Robbins K, Tomalty D, O'Hara R, Tatum C. Transarterial chemoembolization of metastatic colorectal carcinoma with drug-eluting beads, irinotecan (DEBIRI): Multi-institutional registry. . 2009.

12. Martin II RCG, Scoggins CR, Tomalty D, Schreeder M, Metzger T, Tatum C, et al. Irinotecan Drug-Eluting Beads in the Treatment of Chemo-Naive Unresectable Colorectal Liver Metastasis with Concomitant Systemic Fluorouracil and Oxaliplatin: Results of Pharmacokinetics and Phase I Trial. . 2012;16: 1531-1538.

13. Eichler K, Zangos S, Mack MG, Hammerstingl R, Gruber-Rouh T, Gallus C, et al. First human study in treatment of unresectable liver metastases from colorectal cancer with irinotecan-loaded beads (DEBIRI). Int J Oncol. 2012;41: 1213-1220.
