# Supplementary material for: Comparative Effectiveness of Hepatic Artery Based Therapies for Unresectable Colorectal Liver Metastases: A Meta-Analysis
Source: PLoS One. 2015 Oct 8;10(10):e0139940. doi: 10.1371/journal.pone.0139940 (PMC4598149; doi:10.1371/journal.pone.0139940)
Supplement: S4 Table — HAI hepatic arterial infusion; RE radioembolization; TACE transcatheter chemoembolization; HAT hepatic artery based therapy; Sys concurrent systemic chemotherapy; ALP Alkaline Phosphatase. (DOCX) [file pone.0139940.s005.docx]

**Appendix Table 4.** Summary of Specific Grade 3-4 Toxicity (Events per Patient) for HAI, RE and TACE Stratified into Appropriate Subgroups for Comparison

| **Category** | **Grade 3-4 complications % (N= patients evaluable)** | | | | | | | | | |
| --- | --- | --- | --- | --- | --- | --- | --- | --- | --- | --- |
| **All studies** | **Pain** | **Diarrhea** | **Nausea/**  **vomiting** | **Fatigue/**  **asthenia** | **Neutro-penia** | **Increased Bilirubin** | **Elevated**  **ALP** | **Thrombo-**  **cytopenia** | | **Gastric Ulcer** |
| **HAI** | 9.9 (303) | 10.0 (460) | 13.8 (649) | 7.0 (215) | 24.7 (358) | 3.7 (216) | 7.3 (112) | | 2.6 (612) | 2.9 (34) |
| **TACE** | 24.6 (221) | 5.0 (40) | 1.2 (181) | 3.0 (121) | - | 1.0 (121) | 0.8 (276) | | 0 (40) | - |
| **RE** | 2.3 (386) | 0 (72) | 0.6 (342) | 2.3 (349) | 5.2 (19) | 4.3 (299) | 1.6 (490) | | 0 (21) | 1.0 (293) |
| **HAT Alone** |  |  |  |  |  |  |  | |  |  |
| **HAI** | 12.5 (144) | 5.1 (172) | 13.9 (324) | 5.8 (138) | 8.3 (108) | 1.8 (110) | 3.8 (52) | | 1.9 (318) | 3.8 (26) |
| **TACE** | 31.2 (171) | - | 0.9 (141) | 3.0 (121) | - | 1.0 (121) | 2.0 (121) | | - | - |
| **RE** | 1.4 (280) | 0 (72) | 0.7 (280) | 1.4 (280) | - | 4.3 (280) | 2.5 (280) | | - | 1.0 (208) |
| **HAT+ Sys** |  |  |  |  |  |  |  |  | |  |
| **HAI** | 8.8 (384) | 12.4 (258) | 14.3 (303) | 9.2 (77) | 34.9 (228) | 4.8 (83) | 8.1 (37) | | 3.3 (272) | 0 (8) |
| **TACE** | 2.0 (50) | 5.0 (40) | 2.5 (40) | - | - | - | - | | - | - |
| **RE** | 7.7 (65) | - | 0 (21) | 10.0 (40) | 5.2 (19) | 5.3 (19) | 5.2 (19) | | - | 0 (44) |

HAI hepatic arterial infusion; RE radioembolization; TACE transcatheter chemoembolization; HAT hepatic artery based therapy; Sys concurrent systemic chemotherapy; ALP Alkaline Phosphatase
