# Supplementary material for: Comparative Effectiveness of Hepatic Artery Based Therapies for Unresectable Colorectal Liver Metastases: A Meta-Analysis
Source: PLoS One. 2015 Oct 8;10(10):e0139940. doi: 10.1371/journal.pone.0139940 (PMC4598149; doi:10.1371/journal.pone.0139940)
Supplement: S1 Text — (DOCX) [file pone.0139940.s006.docx]

**Search Strategy**

(((("Colorectal Neoplasms"[Mesh] AND "Liver Neoplasms"[Mesh]) AND ("Chemoembolization, Therapeutic"[Mesh] OR "Chemotherapy, Cancer, Regional Perfusion"[Mesh] OR "Embolization, Therapeutic"[Mesh:noexp] OR "Hepatic Artery"[Mesh] OR "Yttrium Radioisotopes"[Mesh] OR "Infusions, Intra-Arterial"[Mesh] OR "Infusion Pumps"[Mesh])) OR ((((Colorectal[All Fields] OR ("colon"[MeSH Terms] OR "colon"[All Fields]) OR ("rectum"[MeSH Terms] OR "rectum"[All Fields])) AND (("liver"[MeSH Terms] OR "liver"[All Fields]) OR hepatic[All Fields])) AND ((hepatic[All Fields] AND ("arteries"[MeSH Terms] OR "arteries"[All Fields] OR "arterial"[All Fields]) AND infusion[All Fields]) OR radioembolization[All Fields] OR radioembolisation[All Fields] OR chemoembolization[All Fields] OR chemoembolisation[All Fields] OR HAI[All Fields])) AND (("neoplasm metastasis"[MeSH Terms] OR ("neoplasm"[All Fields] AND "metastasis"[All Fields]) OR "neoplasm metastasis"[All Fields] OR "metastasis"[All Fields]) OR ("secondary"[Subheading] OR "secondary"[All Fields] OR "metastatic"[All Fields]) OR ("neoplasm metastasis"[MeSH Terms] OR ("neoplasm"[All Fields] AND "metastasis"[All Fields]) OR "neoplasm metastasis"[All Fields] OR "metastases"[All Fields]))) AND ("neoplasms"[MeSH Terms] OR "neoplasms"[All Fields] OR "cancer"[All Fields]))) NOT Case reports
